# Gemcitabine-induced heparanase promotes aggressiveness of pancreatic cancer cells via activating EGFR signaling

**DOI:** 10.18632/oncotarget.16911

**Published:** 2017-04-07

**Authors:** Jin-Wen Song, Ying-Xia Tan, Su-Bo Li, Shi-Kun Zhang, Lu-Ming Wan, Shou-Ping Ji, Hong Zhou, Zhi-Hang Zhou, Feng Gong

**Affiliations:** ^1^ Department of Tissue Engineering, Beijing Institute of Transfusion Medicine, Beijing, China; ^2^ Department of Blood Products and Substitutes, Beijing Institute of Transfusion Medicine, Beijing, China; ^3^ Department of Pathology, The 309th Hospital of People's Liberation Army, Beijing, China

**Keywords:** gemcitabine, pancreatic cancer, HPA1, EGFR, NF-κB

## Abstract

Pancreatic cancer (PC), characterized by aggressive local invasion and metastasis, is one of the most malignant cancers. Gemcitabine is currently used as the standard drug for the treatment of advanced and metastatic PC, but with limited efficacy. In this study, we demonstrated that gemcitabine increased the expression of heparanase (HPA1), the only known mammalian endoglycosidase capable of cleaving heparan sulfate, both *in vitro* and *in vivo*. Furthermore, overexpression of HPA1 in PC cell lines enhanced proliferation and invasion, accompanied with elevated phosphorylation of EGFR. In addition, we showed that the NF-κB pathway mediated the gemcitabine-induced HPA1 expression. Importantly, we found that an HPA1 inhibitor attenuated gemcitabine-induced invasion of PC cells. Finally, we showed that HPA1 was of negative prognostic value for PC patients. Taken together, our results demonstrated that gemcitabine-induced HPA1 promotes proliferation and invasion of PC cells through activating EGFR, implying that HPA1 may serve as promising therapeutic target in the treatment of PC.

## INTRODUCTION

Pancreatic cancer (PC) is the fourth leading cause of cancer related death in the USA with an extremely low 5-year survival of less than 5% [1]. Surgical resection at present offers the only chance for cure. Unfortunately, more than 80% of PC cases are identified as unresectable at diagnosis, due to metastatic or locally advanced tumors, and are subjected to chemotherapy [2]. Gemcitabine has been the standard treatment for advanced and metastatic PC patients [3]. However, most PC patients do not respond well to gemcitabine treatment, and those who do respond may ultimately develop chemoresistance and subsequent disease progression [4]. There is an urgent need to understand the biological mechanisms of disease progression following gemcitabine treatment to improve the prognosis of PC patients.

Heparanase (HPA1) is the only mammalian endoglycosidase capable of cleaving heparan sulfate side chains of heparan sulfate proteoglycans on the cell surface and the extracellular matrix (ECM), leading to the disassembly of the extracellular matrix and facilitation of cell invasion [5]. In addition, HPA1 cleavage activity also induces the mobilization of HS-sequestered growth factors and angiogenic factors, promoting primary tumor growth and angiogenesis [6]. Over-expression of HPA1 has been reported in a variety of tumors including lymphoma, ovarian cancer, prostate cancer, colorectal cancer and pancreatic cancer and is often correlated with decreased survival rates [7–12]. In addition, inhibition of HPA1 has been demonstrated to have an inhibitory effect on cancer invasion, metastasis and angiogenesis [7, 13, 14]. However, the role of HPA1 in treatment with gemcitabine remains unknown. It has been reported that some dosages of gemcitabine promote invasion of PC cells via CXCR4 and CD147, but the mechanisms needs to be further explored [15, 16]. Furthermore, one recent study showed that chemotherapy promoted HPA1 expression in myeloma cells, which induced chemoresistance [17]. Based on the above findings, we envision that gemcitabine may promote the expression of HPA1.

In the present study, we demonstrated that gemcitabine treatment induced the expression of HPA1 via the NF-κB pathway. HPA1 promoted proliferation and invasion of PC cells at least partially through activation of EGFR signaling. A specific HPA1 inhibitor attenuated gemcitabine-induced PC cell invasion. Finally, high expression of HPA1 is of negative prognostic value for PC patients who have received gemcitabine-based chemotherapy. Taken together, our results demonstrated that gemcitabine-induced HPA1 promotes proliferation and invasion of PC cells, implying HPA1 as a promising therapeutic target in the treatment of PC.

## RESULTS

### Gemcitabine induces the expression of HPA1 in PC cells

**Figure 1:**
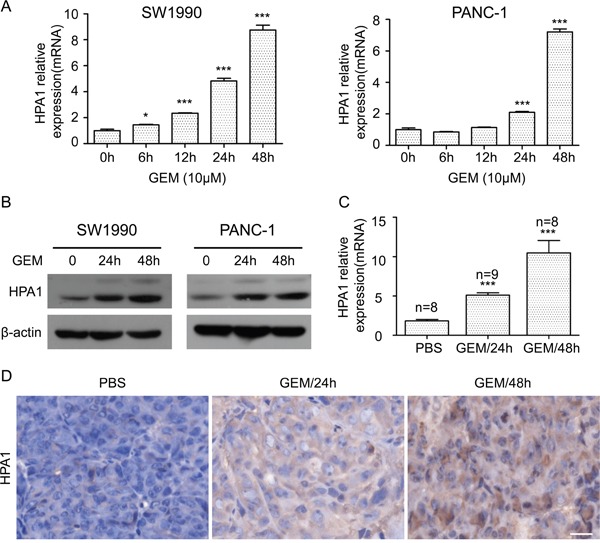
Gemcitabine induces the expression of HPA1 in PC cells *in vitro* and *in vivo* **(A)** The mRNA expression of HPA1 was determined using qRT-PCR in SW1990 (*left panel*) and PANC-1 (*right panel*) cells with or without 10 μM gemcitabine treatment for the indicated times. Results are representative of three independent experiments. **(B)** Western blot analysis was used to determine protein abundance of HPA1 after treatment with 10 μM gemcitabine at the indicated time points. **(C)** BALB/c nude mice bearing SW1990 xenografts were administered a single dose of vehicle (PBS) or gemcitabine (GEM, 50 mg/kg) for either 24 or 48 h. Tumor tissues were obtained and HPA1 mRNA levels were analyzed by qRT-PCR. **(D)** Immunohistochemistry was applied to detect the expression of HPA1 in xenografts following treatment with gemcitabine. Representative images are shown with a scale bar of 20 μm. *p < 0.05, ***p < 0.001 compared with control.

In this study, we first examined the effect of gemcitabine treatment on the growth of SW1990 and PANC-1 cells. The two cell lines were treated with different concentrations of gemcitabine for 48 h with a CCK-8 assay being used to detect proliferation. As shown in Supplementary Figure 1, gemcitabine at the concentration of 10 μM inhibited proliferation of SW1990 and PANC-1 cells by approximately 50%, based on which we decided to treat cell with gemcitabine at this concentration below. As HPA1 plays versatile roles in tumor progression, we then examined the change of HPA1 expression in gemcitabine treated PC cells. The two cell lines were treated with 10 μM gemcitabine for 0, 6, 12, 24, and 48 h, respectively, and qRT-PCR results showed that HPA1 was time-dependently up-regulated (Figure 1A). The induction of HPA1 expression was more evident at 48 h. The elevated protein levels of HPA1 were found at 24 h and 48 h (Figure 1B). We also found that gemcitabine elevated the expression of HPA1 in a dose-dependent manner (Supplementary Figure 1C and 1D). Notably, there was no significant difference in the expression level of HPA1 when the cells were treated with 10 μM or 100 μM gemcitabine. In addition, we found that 5-Fluorouracil induced the expression of HPA1 to a lesser degree, implying the relatively specific effect of gemcitabine on the expression of HPA1 (Supplementary Figure 1E and 1F).

To determine whether gemcitabine induces HPA1 expression *in vivo*, we employed BALB/C nude mice bearing SW1990 flank tumors. The HPA1 mRNA and protein expressions of gemcitabine-treated mice were higher than that of control mice (Figure 1C and 1D). Taken together, these results demonstrate that gemcitabine induces the expression of HPA1 in PC cells both *in vitro* and *in vivo*.

### HPA1 promotes the proliferation and invasion of PC cells

**Figure 2:**
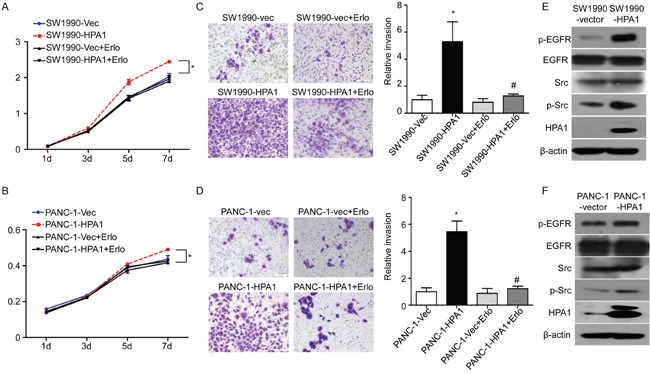
HPA1 promotes the proliferation and invasion of PC cells *in vitro* SW1990 and PANC-1 cells transefected with lentivirus particles containing HPA1 were treated with or without EGFR inhibitor (erlotinib, 5μM). The proliferation of **(A)** SW1990 and **(B)** PANC-1 cells was determined by CCK8 assay. The invasive ability of **(C)** SW1990 and **(D)** PANC-1 cells was measured by transwell invasion assay. SW1990 cells and PANC-1 cells transfected with empty vector were used as control. The expression of p-EGFR and p-Src was upregulated by HPA1 transfection in **(E)** SW1990 cells and **(F)** PANC-1 cells. *p < 0.001 compared with vector, ^#^p<0.001 compared with HPA1 overexpressing cells. Scale bar: 100 μm.

Endogenous HPA1 levels and subcellular location in different PC cell lines were detected by western blotting and immunofluorescence, respectively (Supplementary Figure 2A and 2B). Our results showed that HPA1 was mainly localized in the cytoplasm of PC cells. To further study the role of HPA1 in PC cells, PANC-1 and SW1990 cells were transfected with HPA1 lentiviral activation particles (designated as PANC-1-HPA1 and SW1990-HPA1). Over-expression of HPA1 was confirmed by qRT-PCR (Supplementary Figure 2C and 2D). We then tested the effect of HPA1 on the proliferation of PC cells. Both SW1990-HPA1 and PANC-1-HPA1 cells showed a higher growth rate than the corresponding control cells (Figure 2A and 2B). A cell invasion assay was used to evaluate invasive potential of these cells. SW1990-HPA1 and PANC-1-HPA1 cells invaded to the bottom were significantly increased (approximately 5.3 and 5.5-fold increase, respectively) compared with control (Figure 2C and 2D). In addition, knockdown of HPA1 expression could inhibit invasion of PANC-1 cells (Supplementary Figure 3A and 3B). However, over-expression of HPA1 did not influence the sensitivity to gemcitabine of SW1990 cells (Supplementary Figure 4). To explore the underlying molecular mechanism of HPA1-induced growth and invasion, we detected the expression of epithelial-mesenchymal transition (EMT) markers (E-cadherin, Vimentin, Zeb-1 and Snail) in HPA1 over-expression PC cells. The results showed that over-expression of HPA1 leads to elevated expression of Vimentin, Zeb-1 and Snail and reduced expression of E-cadherin, although the effect was limited (Supplementary Figure 2C and 2D). We then detect the activation of EGFR signaling in HPA1 over-expression PC cells, as the EGFR signaling plays a fundamental role in PC [18, 19]. We found that overexpression of HPA1 significantly promoted phophorylation of EGFR in SW1990 and PANC-1 cells (Figure 2E and 2F). We further detected the downstream signaling following activation of EGFR. The results showed overexpression of HPA1 remarkably increase phosphorylation of Src (Figure 2E and 2F) but not ERK1/2, Stat3 or Akt (Supplementary Figure 3C). In addition, blocking EGFR signaling with erlotinb could abolish HPA1-induced proliferation (Figure 2A and 2B) and invasion (Figure 2C and 2D) of PC cells.

**Figure 3:**
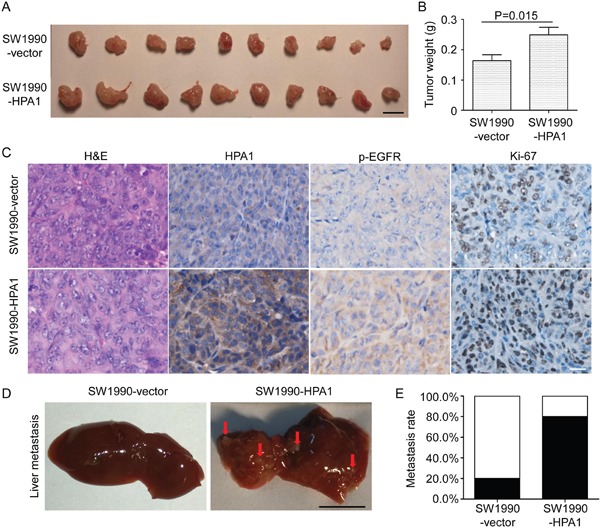
HPA1 promotes the growth and metastasis of PC cells *in vivo* SW1990 cells transfected with HPA1 or empty vector were injected subcutaneously into BALB/c nude mice. Mice were sacrificed 14 days after transplantation, and tumor were excised and weighted. **(A)** Images of xenografts. Scale bar, 1 cm. **(B)** The weight of tumors originated from SW1990-HPA1 cells was larger than that from SW1990-vector cells. **(C)** H&E staining and immunohistochemistry analysis of HPA1, p-EGFR and Ki-67 in the xenografts. The expression of p-EGFR and HPA1 was mainly localized at the cell membrane and cytoplasm. Scale bar, 20 μm. **(D)** Macroscopic appearances of liver from mice injected with SW1990-HPA1 or SW1990-Vector cells. Metastatic nodules were indicated by arrows. Scale bar, 1 cm. **(E)** The metastasis rate was measured and Chi-square analysis showed there was significant difference between SW1990-HPA1 group and SW1990-Vector group.

To further investigate the effect of HPA1 on the proliferation of PC cells *in vivo*, SW1990-HPA1 and SW1990-Vector cells were subcutaneously injected into BALB/c nude mice. Tumors originated from SW1990-HPA1 cells were larger than that of control cells (Figure 3A). The average weight of tumors in the SW1990-HPA1 group was 249±25 mg, whereas the SW1990-Vector group developed smaller tumors with an average weight of 164±19 mg (Figure 3B). Western blot analysis showed higher HPA1 and phosphor-EGFR levels in tumors originated from SW1990-HPA1 cells (Supplementary Figure 5). Immunohistochemistry analysis also validated the over-expression of HPA1, higher Ki-67 positivity and increased phosphorylation of EGFR in tumors originated from SW1990-HPA1 cells (Figure 3C). To investigate whether HPA1 promotes metastasis of pancreatic cancer, SW1990-Vector and SW1990-HPA1 cells were injected into the spleen of BALB/C nude mice to evaluate the metastatic potential of these cells. The results showed that over-expression of HPA1 significantly increased liver metastasis rate from 20% (1/5) to 80% (4/5) (Figure 3D and 3E).

Collectively, these results validate that HPA1 promotes the proliferation, invasion and metastasis of PC cells at least partially via the activation of EGFR signaling.

### The involvement of NF-κB signaling in gemcitabine-induced HPA1 expression

**Figure 4:**
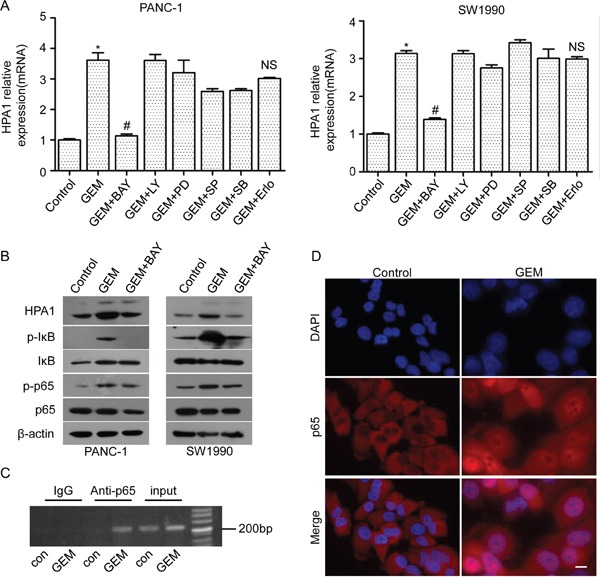
The involvement of NF-κB signaling in gemcitabine-induced HPA1 expression PANC-1 cells and SW1990 cells were pretreated with BAY 11-7082 (10 μM), LY294002 (5 μM), PD98059 (5 μM), SP600125 (10 μM), SB203580 (10 μM) or erlotinib (5 μM) for 2 h and then treated 10 μM gemcitabine for 24 h. **(A)** mRNA levels of HPA1 were detected by qRT-PCR. *p < 0.001 compared with control. ^#^p< 0.001 compared with GEM. NS: non-significant compared with GEM. **(B)** Western blot analysis was used to test the protein levels of HPA1, phospho-IκB, IκB phospho-p65 and p65 in PC cells pretreated with BAY11-7082. **(C)** Chromatins from PANC-1 cells treated with or without gemcitabine (10 μM) for 48h were isolated and subjected to chromatin immunoprecipitation analysis and revealed that gemcitabine promoted p65 binding with HPA1 promoter. **(D)** PANC-1 cells were treated with 10 μM gemcitabine for 48h. Immunofluorescence analysis showed that gemcitabine treatment promoted the nuclear translocation of p65. Scale bar, 20 μm.

To uncover the mechanism of gemcitabine-induced expression of HPA1, we pretreated the PANC-1 and SW1990 cells with BAY11-7082 (inhibitor of NF-κB), LY294002 (inhibitor of PI3K), PD98059 (inhibitor of MEK1/2), SP600125 (inhibitor of JNK), SB203580 (inhibitor of p38) and erlotinib (inhibitor of EGFR) for 2 hours followed by gemcitabine treatment. The expression of HPA1 was then determined by qRT-PCR. The results showed that BAY11-7082 significantly reduced gemcitabine-induced HPA1 expression while other inhibitors had limited effect (Figure 4A). BAY11-7082 also reduced gemcitabine-induced HPA1 expression at the protein levels (Figure 4B). Consistent with previous study [20, 21], we found that binding of p65 to the promoter region of HPA1 gene was dramatically increased following treatment with gemcitabine using a ChIP assay (Figure 4C). Moreover, immunofluorescence staining revealed that gemcitabine induced NF-κB activation by promoting the nuclear translocation of p65 (Figure 4D). Stimulation with TNF-α was used as a positive control (Supplementary Figure 6). Thus, we conclude that gemcitabine treatment promotes HPA1 expression via NF-κB pathway.

### HPA1 inhibitor attenuated gemcitabine-induced invasiveness of PC cells *in vitro*

**Figure 5:**
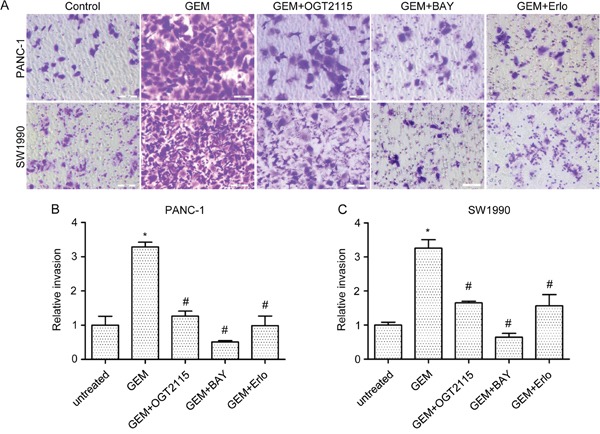
HPA1 inhibitor attenuates gemcitabine-induced invasiveness of PC cells *in vitro* **(A)** *In vitro* cell invasion of PANC-1 and SW1990 cells treated with gemcitabine (10μM, 24 hours) alone or in combination with OGT2115 (1 μM), BAY 11-7082 (10 μM) or erlotinib (5 μM). Invaded cells were stained with 0.1% crystal violet and examined by light microscopy **(B)**. Invasion ability of **(B)** PANC-1 and **(C)** SW1990 cells was determined by counting the cells invaded to the lower chamber. Scale bar, 100 μm. *p < 0.001 compared with untreated.^#^p< 0.001 compared with GEM.

Other studies have shown that gemcitabine treatment may stimulate PC cell invasiveness [16]. We then investigated whether gemcitabine-induced invasiveness is associated with the gemcitabine-induced increase in HPA1 expression. As expected, gemcitabine enhanced the invasion of both PANC-1 (3.3-fold) and SW1990 (3.2-fold) cells and this enhancement was attenuated in the presence of a specific HPA1 inhibitor, OGT 2115 (Figure 5). As mentioned above, gemcitabine-induced activation of NF-κB signaling increased the expression of HPA1, which further promoted the phosphorylation of EGFR. We then tested whether BAY 11-7082 and erlotinib could inhibit gemcitabine-induced invasion. The results showed that both BAY 11-7082 and erlotinib attenuated gemcitabine-induced invasion of PC cells (Figure 5).

In addition, we also observed that OGT 2115 augmented the inhibitory growth effect of gemcitabine in SW1990 cells (Supplementary Figure 7). These results indicate that inhibition of HPA1 attenuates the gemcitabine-induced invasion of PC cells and enhances the efficiency of gemcitabine.

### HPA1 is of negative prognostic value for PC patients

**Figure 6:**
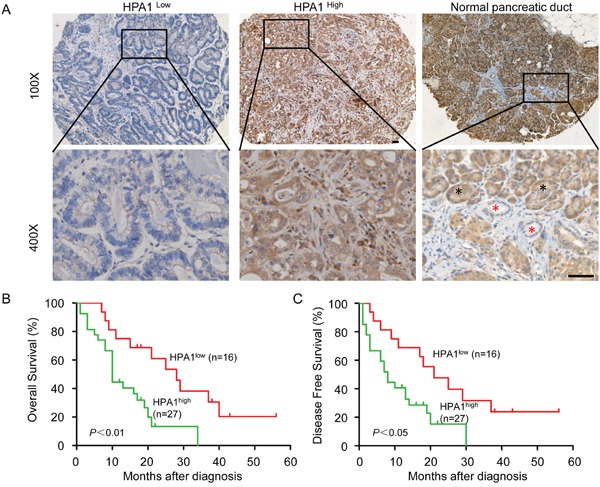
HPA1 is of negative prognostic value for PC patients **(A)** Immunohistochemistry analysis was performed on paraffin-embedded tissue microarray of 43 patients with pancreatic cancer using the primary antibody against HPA1. Representative images of immunohistochemical staining showing low expression of HPA1 in well differentiated PC tissue (*left panel*) and high expression of HPA1 in poorly differentiated PC tissue (*middle panel*). HPA1 staining in normal pancreatic duct cells were indicated by red asterisk and pancreatic acinar cells were indicated by black asterisk (*right panel*). **(B)** Kaplan-Meier analysis of overall survival of PC patients with high or low expression of HPA1. **(C)** Kaplan-Meier analysis of disease free survival of PC patients with high or low expression of HPA1. Scale bar, 50 μm.

Given the above results, we further examined the clinical significance of HPA1 expression in primary human PC using a tissue microarray. To this end, samples from 43 cases of PC patients who received gemcitabine-base chemotherapy were collected and examined by immunohistochemistry. Positive HPA1 staining was mainly observed in cytoplasm. High-expression of HPA1 was detected in 62.8% (27/43) of the tumor tissues and the representative images of HPA1 staining in tumors and normal pancreatic ducts were shown in Figure 6A.

We then analyzed the relationship between HPA1 expression levels and the clinical parameters. Consistent with a previous study showing that high HPA1 mRNA levels were significantly associated dedifferentiation in PC [22], we showed that high HPA1 expression was correlated with low differentiation degree by immunohistochemistry (Table 1). In addition, HPA1 expression was significantly correlated with p65 expression (*p*=0.006), further suggesting that HPA1 expression might be regulated by NF-κB signaling. Representative immunohistochemistry images of p65 and HPA1 staining were shown in Supplementary Figure 8. Positive correlation between the expression level of HPA1 and phosphor-EGFR though without significance. Kaplan-Meier survival analysis demonstrated that the overall survival or disease-free survival was significantly shorter in patients with high expression of HPA1 (Figure 6B and 6C). These results showed that the HPA1 predicts poor prognosis for patients who received gemcitabine-based chemotherapy.

**Table 1:** The correlation between the HPA1 expression level and the clinicopathological parameters in PC samples from 43 patients

Clinicopathologic features		No. of patients (%)	HPA1 expression status		*P* value
			Low (n=16) No.patient (%)	High (n=27) No.patient (%)	
p-EGFR					
	Negative	28(65.1)	13(46.4)	15(53.6)	0.087
	Positivie	15(34.9)	3(20.0)	12(80.0)	
p65					
	Low	18(41.9)	11(68.8)	7(25.9)	0.006
	High	25(58.1)	5(31.2)	20(74.1)	
Gender					
	Male	29(67.4)	10(62.5)	19(70.4)	0.594
	Female	14(32.6)	6(37.5)	8(29.6)	
Age					
	≤58	19(44.2)	11(68.8)	8(29.6)	0.013
	>58	24(55.8)	5(31.2)	19(70.4)	
Tumor size(cm)					
	≤3.2	27 (62.8)	9(56.3)	18(66.7)	0.495
	>3.2	16(37.2)	7(43.7)	9(33.3)	
Differentiation degree					
	Well	7(16.3)	5(31.3)	2(7.4)	0.045
	Moderate	23(53.5)	9(56.2)	14(51.9)	
	Poor	13(30.2)	2(12.5)	11(40.7)	
T-stage					0.417
	T1	8(18.6)	3(18.8)	5(18.5)	
	T2	13(30.2)	3(18.8)	10(37.0)	
	T3	22(51.2)	10(62.5)	12(44.4)	
Lymph node metastasis					0.594
	Negative	29(67.4)	10(62.5)	19(70.4)	
	Positive	14(32.6)	6(37.5)	8(29.6)	
Distant metastasis					0.436
	Negative	42(97.7)	16(100)	26(96.3)	
	Positive	1(2.3)	0(0)	1(3.7)	
TNM stage					0.763
	I	18(41.8)	6(37.5)	12(44.4)	
	II	11(25.6)	4(25.0)	7(25.9)	
	III	13(30.2)	6(37.5)	7(25.9)	
	IV	1(2.3)	0(0.0)	1(3.7)	

## DISCUSSION

In spite of much progress being made in the treatment of solid tumors, the prognosis for PC remains poor. Most PC patients are diagnosed with locally advanced diseases or metastatic diseases, and miss the opportunity for surgical resection [23]. Moreover, those who are eligible for resection may ultimately develop local recurrence of pancreatic tumor, metastasis or both [24, 25]. Thus, they are all subjected to chemotherapy. Metastasis is a common feature of PC, an important step of which is breaking through the extracellular matrix [26]. Heparan sulfate proteoglycans (HSPGs) are essential components of the extracellular matrix, basement membrane and cell surface [27]. HPA1, the only known mammalian endoglycosidase capable of cleaving heparan sulfate side chains of HSPGs, is an versatile protein affecting multiple events in the context of tumors, including tumor invasion, metastasis and angiogenesis [28]. Elevated HPA1 expression has also been well documented in a variety of tumors, including PC [12, 29]. Our present data shows that HPA1 promotes PC cell proliferation and invasion via activating EGFR signaling. Moreover, high expression of HPA1 is correlated with poor differentiation and predicts poor clinical outcomes in PC patients.

Gemcitabine is a nucleoside analog that has been used as the standard care drug for locally advanced and metastatic pancreatic cancer [30]. Gemcitabine inhibits DNA synthesis by accumulation of difluorodeoxycitidine triphosphate, which competes with deoxycitidine triphosphate for incorporation into DNA [31]. However, other studies reported that there were some undesirable effects in response to gemcitabine treatment. For example, gemcitabine promoted invasiveness and stemness, as well as triggered angiogenesis-promoting molecular signals of PC cells [15, 16, 32, 33]. Our results demonstrated that gemcitabine could promote HPA1 expression in PC cells (Figure 1). On the other hand, 5-FU, another commonly used drug for the treatment of PC, failed to induce HPA1 expression (Supplementary Figure 1E and 1F). 5-FU is an analogue of uracil and the metabolites of 5-FU disrupt RNA synthesis and the action of thymidylate synthase [34]. We propose that the different effect of gemcitabine and 5-FU may due to their ability to activate NF-κB signaling, as gemcitabine but not 5-FU was documented to activate NF-κB signaling in PC [15, 35]. Activating of NF-κB by gemcitabine is time-dependent [15], giving a reason why HPA1 induction is prominent at relative late time points (48h) or even later (72h and 96h, data not shown).

We also showed that gemcitabine-induced invasion could be attenuated by HPA1 or EGFR inhibitor (Figure 5). Gemcitabine has been reported to promote PC cells invasion via upregulation of CXCR4 and CD147 [15, 16]. There are some connection among HPA1, CXCR4 and CD147: both CXCR4 and HPA1 are downstream targets of NF-κB signaling; while both CD147 and HPA1 could activate EGFR signaling pathway. Our results suggest that some undesirable effects of gemcitabine treatment might be related with up-regulation of HPA1 and a combination of HPA1 inhibitors with gemcitabine might be beneficial in the treatment of PC. Indeed, some HPA1 inhibitors have been produced. For example, M402 is currently in a phase I/II trial in combination with nab-paclitaxel and gemcitabine for the treatment of metastatic PC [36]. Actually, other anti-tumor drugs, such as tamoxifen, bortezomib and carfilzomib, have recently been demonstrated to induce the expression of HPA1 in breast cancer or myeloma [37, 38]. It should be noted that radiotherapy has also been reported to promote PC cell invasion through elevating the expression of HPA1 [39]. Several researchers have observed that HPA1 reduced the chemotherapeutic sensitivity of tumor cells [17, 40, 41]. However, we found that over-expression of HPA1 did not influence the sensitivity to gemcitabine of SW1990. We speculate that this is due to a cell type specific effect of HPA1 or the relatively low sensitivity of SW1990 cells. PC patients showed a low response rate to gemcitabine because of intrinsic chemoresistance or acquired resistance developed after repeated exposure. Several mechanisms have been revealed for gemcitabine resistance in PC, such as mechanisms related to gemcitabine metabolism and aberrant activation of NF-κB signaling [30]. We also found that HPA1 promotes activation of the EGFR signaling, which plays essential role in the progression of PC [18, 19].

NF-κB is a ubiquitous transcription factor regulated by many stimuli, including hypoxia, growth factors, chemotherapy drugs, UV light and others [42, 43]. NF-κB is a dimer composed of various combinations of five mammalian Rel proteins, namely, p65/RelA, c-Rel, RelB, NFκB1/p50, and NFκB2/p52 [44]. The most common form is a dimer composed of p65 and p50. Increased nuclear location of p65 is an indicator of NF-κB activation. Consistent with previous studies, we found that gemcitabine activated NF-κB signaling in PC cells [15, 45]. Our results showed that pretreatment with BAY11-7082 could attenuate gemcitabine-induced HPA1 expression. We showed that gemcitabine promoted binding of p65 with the promoter of the HPA1 gene in PANC-1 cells, which is consistent with other groups [20, 21]. Interestingly, we found that HPA1 was also expressed in stromal cells in PC tissues. It has been reported that HPA1 stimulates macrophage activation, while macrophages induce production and activation of latent HPA1 contributed by the colon epithelium, generating a vicious cycle that power colitis and the associated tumorigenesis [46]. In addition, fibroblasts may also be involved in activation, processing and trafficking of extracelluar HPA1 [47]. As tumor microenvironment has been recognized as a pivotal driver of cancer progression, the role of HPA1 in the tumor stromal cells needs to be determined.

In summary, the present study revealed that gemcitabine-induced HPA1 promoted invasion and metastasis of pancreatic cancer cells via activating EGFR signaling. We also demonstrated that gemcitabine promoted HPA1 expression via activating the NF-κB pathway. Therefore, a specific HPA1 inhibitor together with gemcitabine might be a novel strategy for the treatment of patients with PC.

## MATERIALS AND METHODS

### Cell lines and patient samples

The PC cell lines SW1990, PANC-1, Bxpc-3, CFPAC-1, patu8988, and Aspc-1 were obtained from American Type Culture Collection and were maintained in RPMI-1640 medium or DMEM supplemented with 10% fetal bovine serum at 37°C in a humidified atmosphere of 95% air and 5% CO_2_.

Tumor tissues from forty-three cases of pancreatic cancer patients from 2011 to 2014 at the 309th hospital of PLA were collected. The patients received gemcitabine-based chemotherapy after surgery. The diagnosis of pancreatic cancer was made independently by at least two histopathologists. This study was carried out in accordance with the principles of the Helsinki Declaration and approved by the Ethical Committee of 309th hospital of PLA (No. 2015013). Written informed consent was obtained from all patients.

### Antibodies and reagents

Gemcitabine, LY294002, PD98059, SP600125, SB203580 and erlotinib were purchased from Selleck Chemicals (Houston, TX). Puromycin, Blasticidin S HCl and Hygromycin B were obtained from Santa Cruz Biotechnology (CA, USA). Monoclonal mouse anti-human β-actin antibodies were purchased from Sigma-Aldrich (St Louis, MO). Monoclonal mouse anti-human HPA1 antibodies were purchased from proteintech^TM^ (Chicago, IL). Alexa Fluor 488-labeled goat anti-mouse IgG, Cy3-labeled goat an-rabbit IgG and BAY 11-7082 was purchased from Beyotime (Shanghai, China). Polyclonal rabbit anti-human p65, phospho-p65 (Ser536), EGFR, phospho-EGFR (Tyr1068), Src, phospho-Src (Tyr416), ERK1/2, phosphor-ERK1/2, Stat3, phosphor-Stat3 (Tyr705), Akt and phosphor-Akt (Ser473) were purchased from Cell Signaling Technology (Beverly, MA).

### RNA extraction and qRT-PCR

Total RNA was isolated with TRIzol regent (Invitrogen, Carlsbad, CA) and reverse transcribed to cDNA using PrimerScript^TM^ RT master mix (Takara, Dalian, China) according to the manufacture instructions. Briefly, the 20μl reaction system included 1μg total RNA and 4μl 5X PrimerScript Buffer. PCR was performed in a CFX96 real-time PCR system (Bio-Rad, Richmond, CA) using SYBR Green (Takara, Dalian, China). Primers used in this study were listed in Supplementary Table 1.

### Immunofluorescence staining

Cells were seeded into 24-well plates containing glass coverslips on the bottom. Cells were fixed in 4% paraformaldehyde for 30 min and permeabilized with 0.1% Triton-X for 10 min. Then, cells were blocked and incubated with the anti-HPA1 antibody for 1 h at room temperature. Fluorescence-conjugated secondary antibodies were incubated for 1 h at room temperature. Cell nucleus was stained with hoechest 33342 for 5 min. Images were captured with an inverted fluorescence microscope (PerkinElmer, Norwalk, CT).

### Western blot

Total proteins from cell were extracted in a lysis buffer (Beyotime, Shanghai, China). Proteins were subjected to SDS-PAGE and transferred to PVDF membranes (Darmstadt, Germany). Membranes were incubated overnight at 4°C with antibody against HPA1, phosphor-p65, p65 or β-actin, followed by HRP-conjugated secondary antibody. Immunoreactivity was visualized using chemiluminescence ECL (Pierce, Rockford, IL).

### CCK8 assay

Cell proliferation was determined using CCK8 assay kit (Beyotime, Shanghai, China). Cells were seeded in 96-well plates at 2000 cells/well. 10μl of the CCK8 solution was added to each well and incubated for 1 h. The absorbance was measured at 450 nm using a SpectraMax M5 plate reader (Molecular Devices, Sunnyvale, CA).

### Chromatin immunoprecipitation (ChIP) assay

PANC-1 cells were harvested and CHIP assay was performed using a CHIP assay kit (Cell Signaling, Beverly, MA). Anti-p65 or rabbit anti-IgG antibodies were used for precipitation, and the immunoprecipitates were collected with Agarose Beads. The primers used for amplifying the NF-κB binding site in HPA1 promoter were as follows: forward, 5’-AATGTTGAGCAACATCACAATAC-3’; and reverse, 5’-GTG TCAGAATCGGGATGTGAAGTGTC-3’.

### Cell transfection

Cells were transfected with HPA1 lentiviral activation particles or control lentiviral activation particles (Santa Cruz, CA, USA) in the presence of 8μg/ml polybrene. Stable transfected cells were selected with 2μg/ml puromycin, 400μg/ml Hygromycin B and 5 μg/ml Blasticidin S HCl.

For RNA interference, HPA1 siRNA (target: CTCGAAGAAAGACGGCTAA) was designed and synthesized (Ribobio, GuangZhou, China) as follows: 5’-CUCGAAGAAAGACGGCUAAtt-3’ (sense) and 5’-UUAGCCGUCUUUCUUCGAGtt-3’ (antisense). The sequence for negative control siRNA (NC): 5’-AGCAUCGUACGUAGGCCAGtt-3’ (sense), and 5’-CUGGCCUACGUACGAUG CUtt-3’ (antisense). siRNA were transfected by Lipofectamine 2000 (Invitrogen, CA, USA) according to the manufacturer's instructions.

### Cell invasion assay

PANC-1 (2×10^4^) and SW1990 (1×10^5^) cells were suspended in 100μl serum-free medium and seeded into the upper chamber with 8 μm pore polycarbonate membranes precoated with Matrigel Basement Membrane Matrix (BD Biosciences, Bedford, MA). The lower chamber was filled with 600μl of 1640 medium supplemented with 10% FBS. After 24 hours of incubation, cells remaining in the upper membrane surface were removed with a cotton swab, whereas invaded cells were fixed and stained with 0.5% crystal violet, and counted by examining 5 randomly selected fields.

### Animal experiments

All animal experiments were performed in accordance with Institutional Animal Care and Use Committee approved protocols. For subcutaneous xenograft mouse model, 2×10^5^ SW1990 cells transfected with HPA1 or vector were suspended in serum-free-RPMI/Matrigel mixture (1:1 volume) and then injected subcutaneously into the back of BALB/c nude mice. After 3 weeks, mice were sacrificed and tumors were harvested. For *in vivo* liver metastasis assay, 1×10^6^ SW1990 cells transfected with HPA1 or vector were suspended in phosphate-buffered saline and then injected into the spleen of BALB/c nude mice. Mice were sacrificed and liver metastasis was analyzed 5 weeks after intrasplenic injection. To determine the effect of gemcitabine on HPA1 expression *in vivo*, mice bearing SW1990 xenografts were administered with gemcitabine (50 mg/kg) or PBS by intraperitoneal injection. After 24 or 48 h, the mice were sacrificed and tumors were harvested for *ex vivo* analysis.

### Immunohistochemical staining

Following deparaffinization and rehydration, tumor sections of 4 μm were incubated in 0.3% H_2_O_2_ in methanol for 30 minutes at 37°C to block endogenous peroxidase. The sections were then boiled in 10 mmol/L citrate buffer (pH 6.0) for 15 minutes in a microwave oven. The antibodies against HPA1, phosphor-EGFR, p65 or Ki-67 (MXB Biotechnologies, China) were added, and the sections were incubated at 4°C overnight. The sections were visualized using the diaminobenzidine solution (MXB Biotechnologies, China) and were then lightly counterstained with hematoxylin. Sections without incubation with primary antibody served as negative controls. The intensity of staining (brown color) was semi-quantitatively scored as follows: 1, weak; 2, medium; 3, strong; and 4, very strong. The percentage of maximally stained tumor cells in each section was recorded (0, <5%; 1, 5-30%; 2, 30–50%; 3, >50%). High expression of HPA1 was defined as a combined score for the intensity and area of staining that was ≥4. The results were verified by two histopathologists independently.

### Statistical analyses

Statistical analyses were performed using Graphpad Prism 5.0 (Graphpad, San Diego, CA) using Student's *t*-tests or ANOVA test. The results are expressed as the mean ± SD except for where noted. P value less than 0.05 was considered statistically significant.

## SUPPLEMENTARY MATERIALS FIGURES AND TABLE


