# Supplementary material for: Gemcitabine-induced heparanase promotes aggressiveness of pancreatic cancer cells via activating EGFR signaling
Source: Oncotarget. 2017 Apr 7;8(35):58417–29. doi: 10.18632/oncotarget.16911 (PMC5601663; doi:10.18632/oncotarget.16911)
Supplement: Supplementary file 1 [file oncotarget-08-58417-s001.pdf]

Gemcitabine-induced heparanase promotes aggressiveness of pancreatic cancer cells via activating EGFR signaling

SUPPLEMENTARY FIGURES AND TABLE

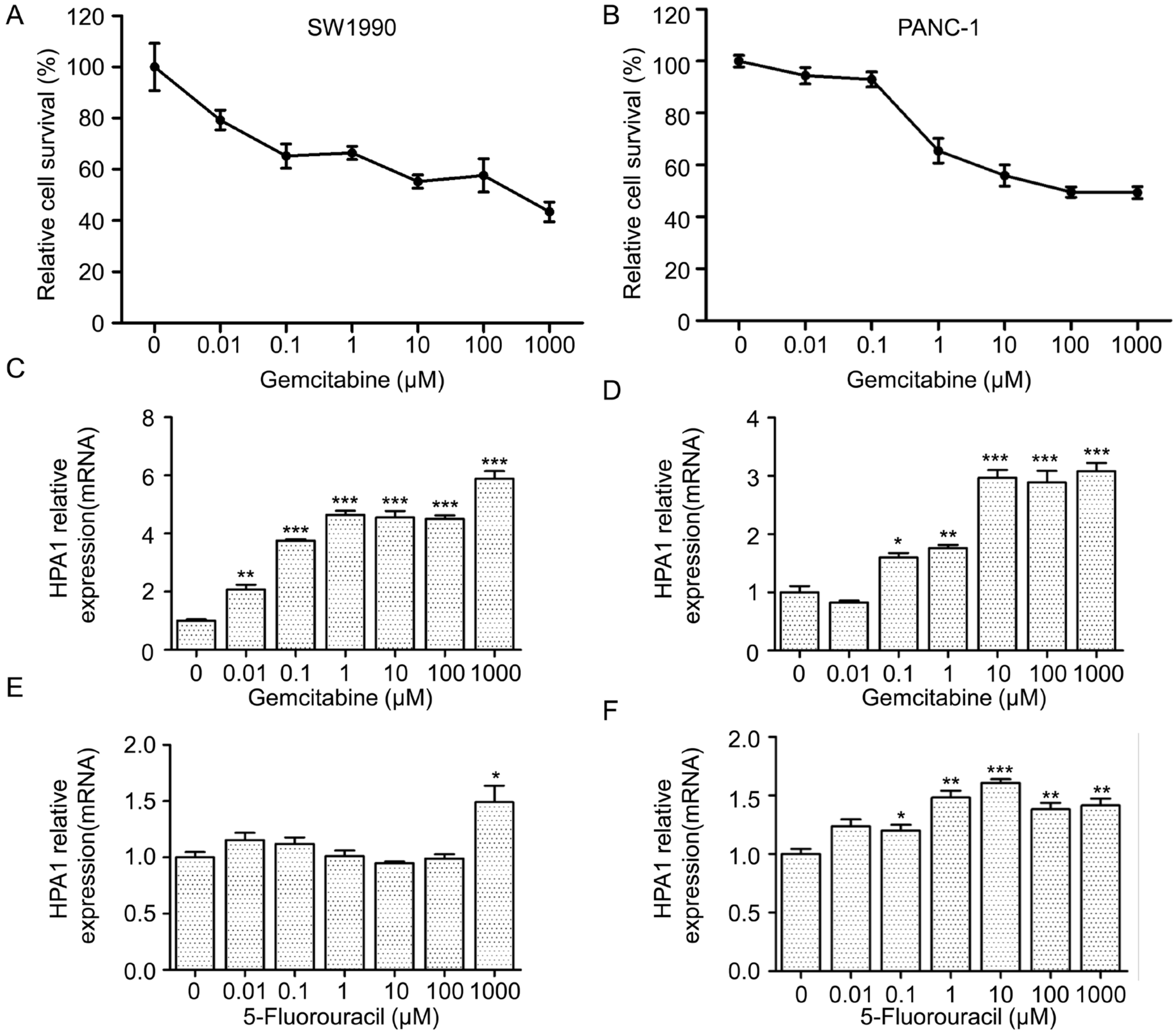

Supplementary Figure 1: Gemcitabine and 5-Fluorouracil promote the expression of HPA1 *in vitro*. (A) SW1990 and **(B)** PANC-1 cells were treated with 0.1–1000 μM gemcitabine for 48 h, and the relative survival rate was detected by CCK-8 assay. The mRNA expression of HPA1 in **(C)** SW1990 and **(D)** PANC-1 cells were determined by qRT-PCR after various concentrations of gemcitabine treatment for 48 h. The mRNA expression of HPA1 in **(E)** SW1990 and **(F)** PANC-1 cells were determined by qRT-PCR after various concentrations of 5-Fluorouracil treatment for 48 h. *p < 0.05, ***p < 0.001.

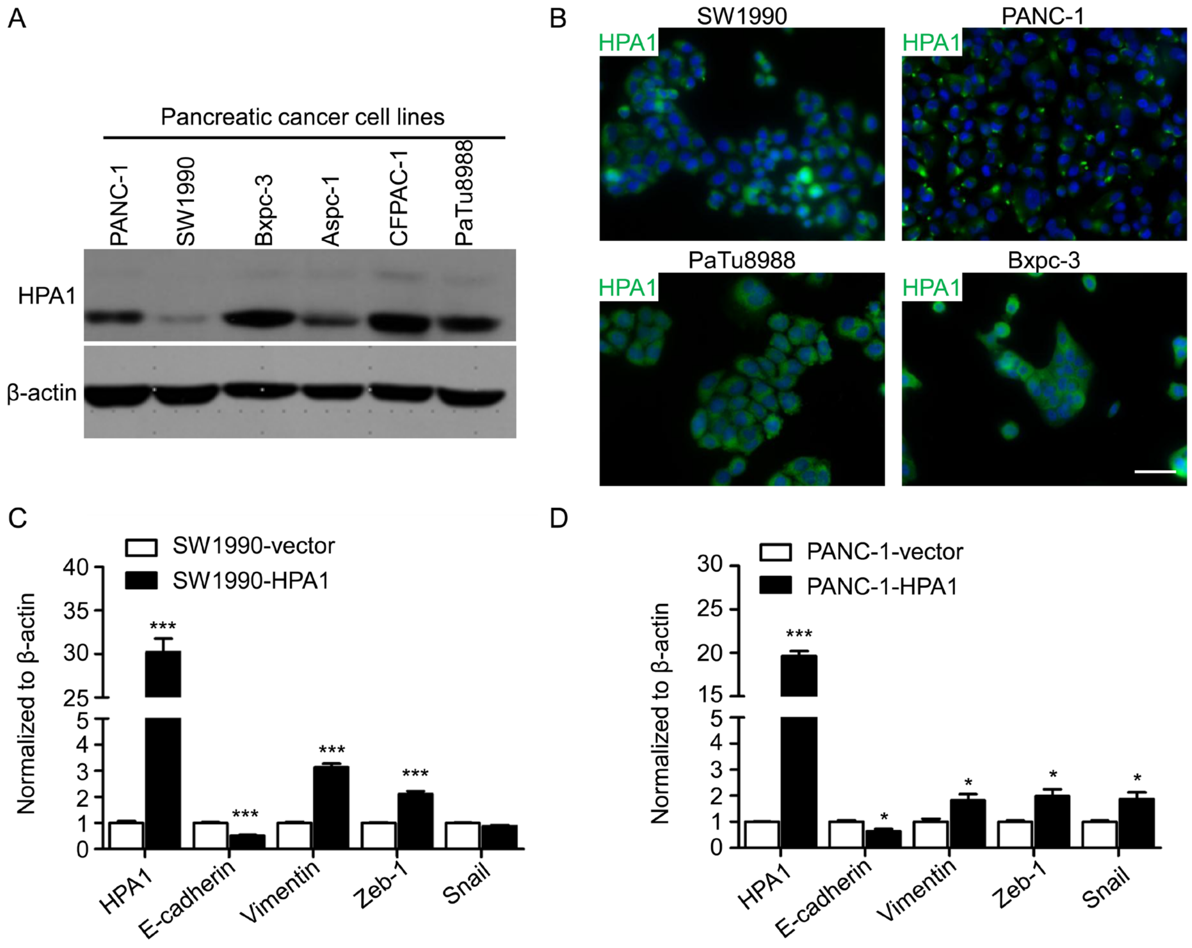

Supplementary Figure 2: The expression and location of HPA1 in PC cell lines. **(A)** Western blot analysis of HPA1 expression in human pancreatic cancer cell lines. **(B)** Immunofluorescence was used to determine the localization of HPA1 in PC cell lines. **(C)** The mRNA expression of HPA1, E-cadherin, Vimentin, Zeb-1 and Snail were determined using qRT-PCR in HPA1 overexpressing **(C)** SW1990 and **(D)** PANC-1 cells. Scale bar, 50 µm. $*p < 0.05$, $**p < 0.01$, $***p < 0.001$.

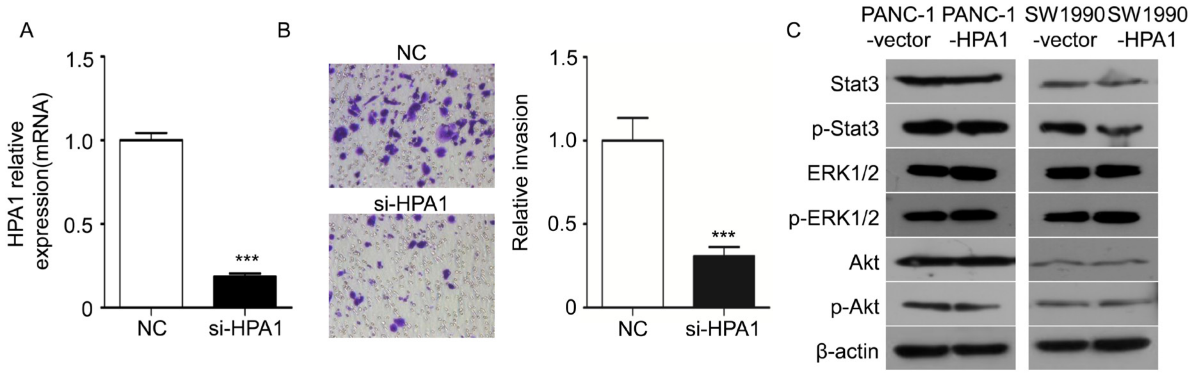

Supplementary Figure 3: Knock-down of HPA1 inhibit invasion of PANC-1 cells. PANC-1 cells were transfected with negative control (NC) siRNA and siRNA targeting HPA1 (si-HPA1). **(A)** Expression of HPA1 detected by qRT-PCR. **(B)** Invasive ability was determined by transwell invasion assay. **(C)** The expression of p-Stat3, Stat3, p-ERK1/2, ERK1/2, p-Akt and Akt in PANC-1 and SW1990 cells overexpressing HPA1 were determined by western blot.

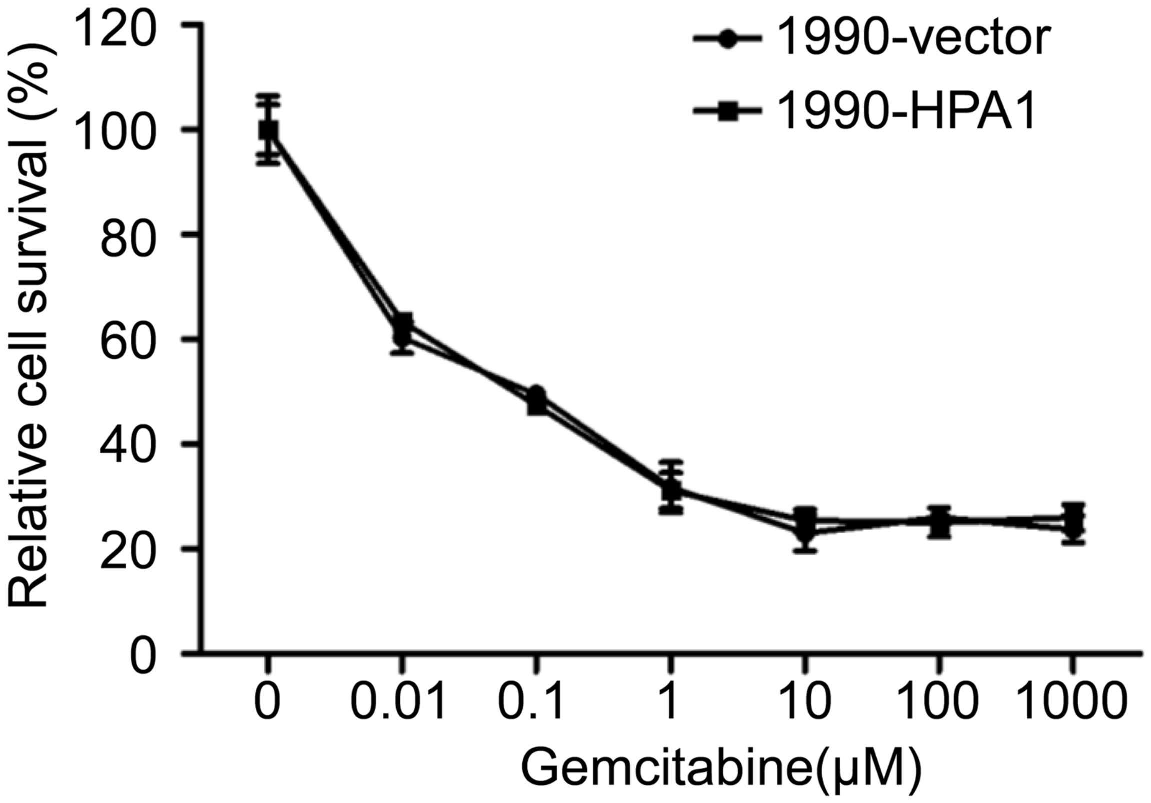

Supplementary Figure 4: Comparison of gemcitabine sensitivity between SW1990-vector and SW1990-HPA1 cells. SW1990-vector and SW1990-HPA1 cells were treated with 0.1–1000 μM gemcitabine for 48 h and the relative survival rate was determined by CCK-8 assay.

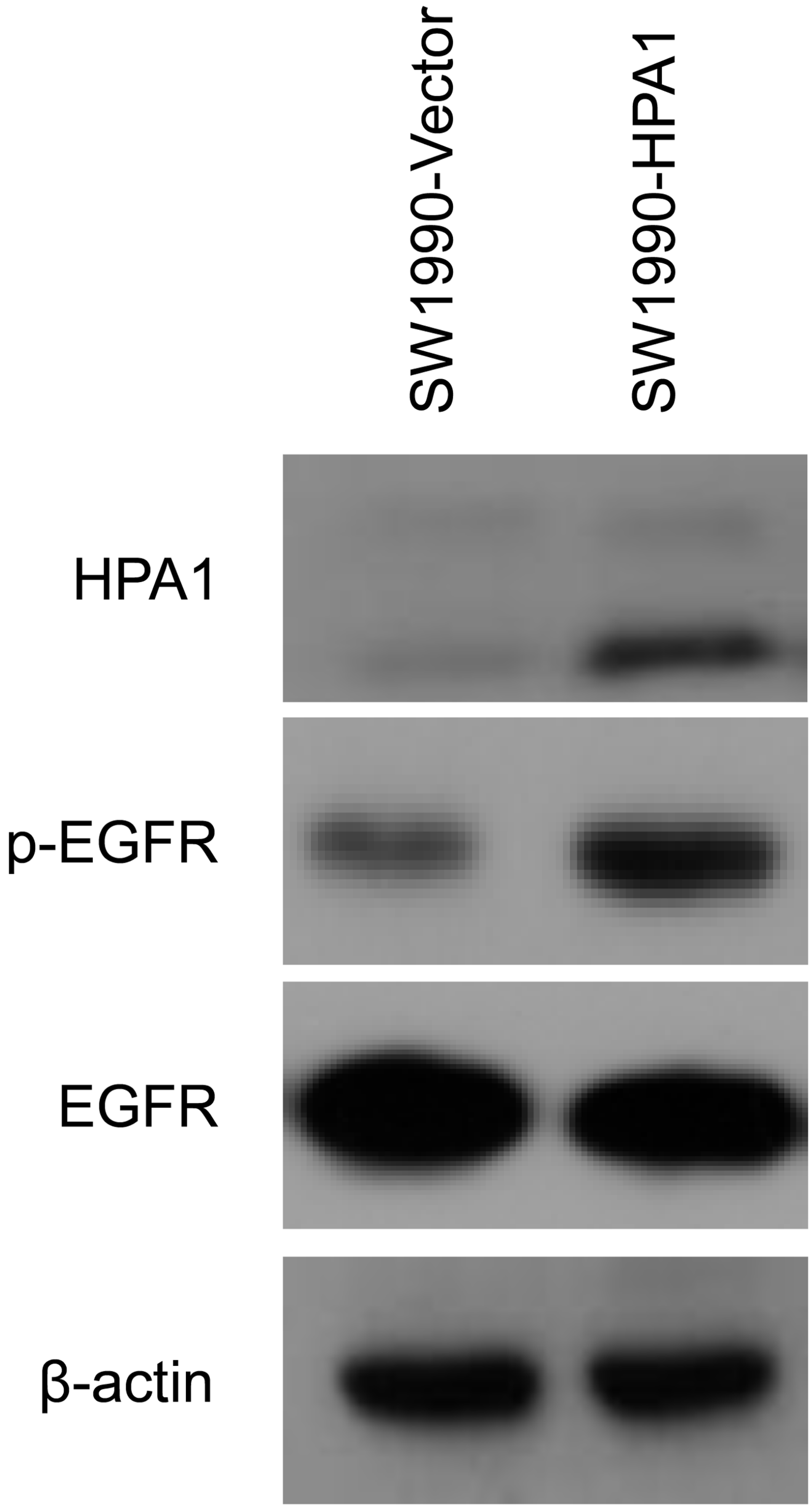

Supplementary Figure 5: Validation of HPA1 expression of the xenografts. Western blot analysis was used to detect HPA1, p-EGFR and EGFR expression in SW1990-vector and SW1990-HPA1 xenografts.

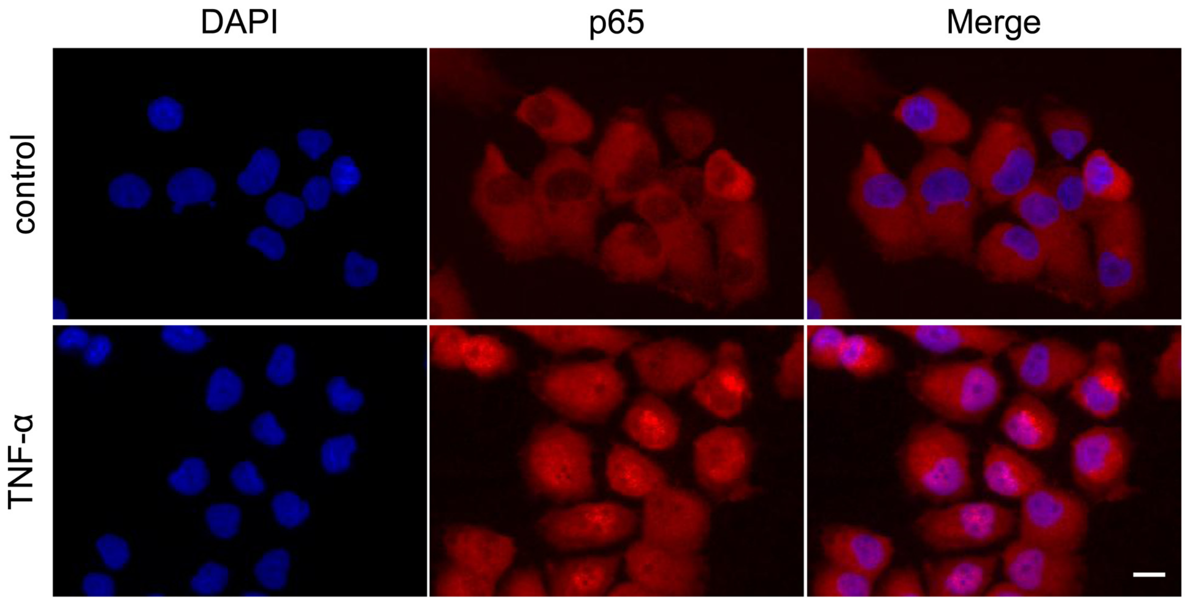

Supplementary Figure 6: TNF-α activated NF-κB signaling pathway. PANC-1 cells were treated with TNF-α (30 ng/ml) for 1 h and immunofluorescence analysis showed that TNF-α promoted nuclear translocation of p65. Scale bar, 20 µm.

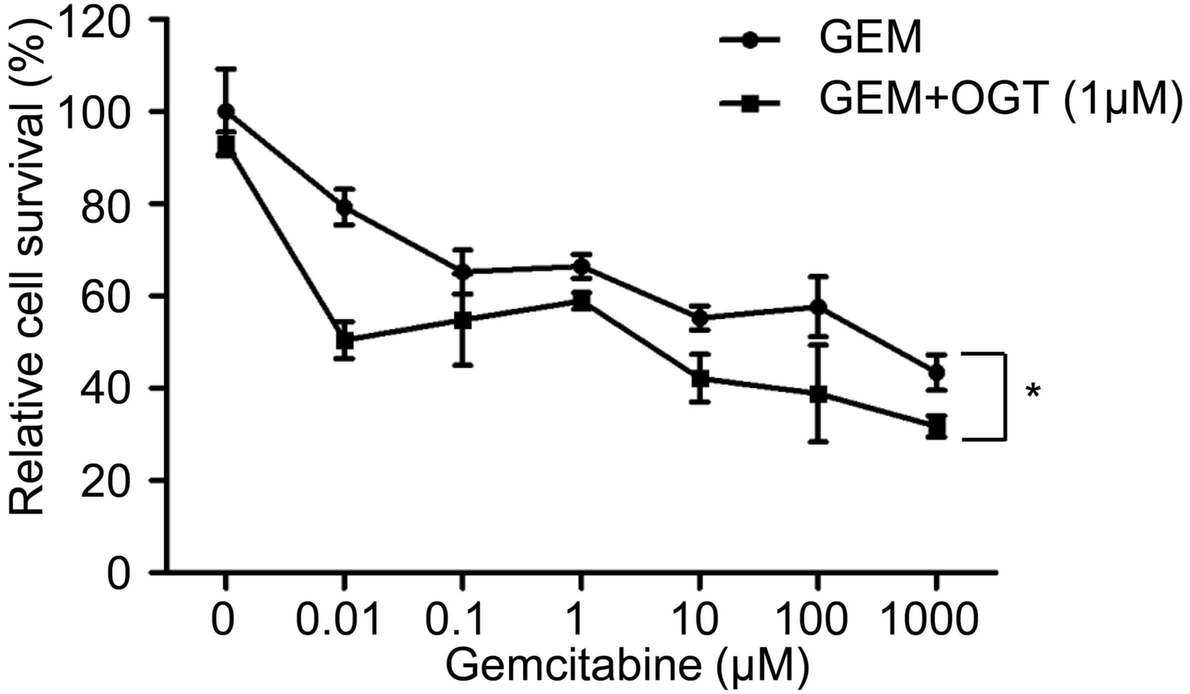

Supplementary Figure 7: Synergistic effects of OGT2115 and gemcitabine co-treatment on SW1990 cells. SW1990 cells were treated with different concentrations of gemcitabine alone or in combination with 1 μM OGT2115. Relative cell survival was measured by CCK-8 assay. *$p < 0.05$.

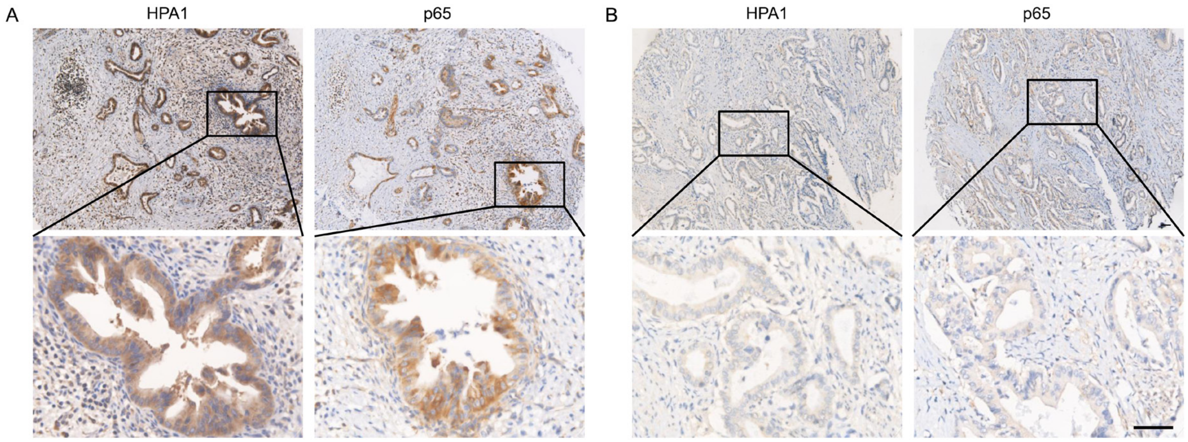

Supplementary Figure 8: Immunohistochemistry staining of p65 and HPA1. (A) Representative immunohistochemistry images with intense HPA1 (*left panel*) and p65 (*right panel*) staing. **(B)** Representative immunohistochemistry images with weak HPA1 (*left panel*) and p65 (*right panel*) staing. Scale bar, 50 μm.

Supplementary Table 1: The primers used in the present study

Primer's name	Sequence	Product length
HPA1-F	TACCTTCATTGCACAAACACTG	88
HPA1-R	ACTTGGTGACATTATGGAGGTT	
β-actin-F	CCTGTACGCCAACACAGTGC	211
β-actin-R	ATACTCCTGCTTGCTGATCC	
E-cadherin-F	CCCACCACGTACAAGGGTC	176
E-cadherin-R	ATGCCATCGTTGTTCACTGGA	
Vimintin-F	TACAGGAAGCTGCTGGAAGG	104
Vimintin-R	ACCAGAGGGAGTGAATCCAG	
Zeb-1-F	ACCCTTGAAAGTGATCCAGC	142
Zeb-1-R	CATTCCATTTTCTGTCTTCCGC	
Snail-F	ACCACTATGCCGCGCTCTT	115
Snail-R	GGTCGTAGGGCTGCTGGAA	
